# The effect of telephone-based interpersonal psychotherapy for the treatment of postpartum depression: study protocol for a randomized controlled trial

**DOI:** 10.1186/1745-6215-13-38

**Published:** 2012-04-19

**Authors:** Cindy-Lee Dennis, Paula Ravitz, Sophie Grigoriadis, Melissa Jovellanos, Ellen Hodnett, Lori Ross, John Zupancic

**Affiliations:** 1 University of Toronto, Lawrence S. Bloomberg Faculty of Nursing, 155 College Street, Suite 130, Toronto, ON, M5T 1P8, Canada; 2 Women’s College Hospital, Women’s College Research Institute, 790 Bay Street, Toronto, ON, Canada; 3 Department of Psychiatry, Mount Sinai Hospital, Joseph and Wolf Lebovic Health Complex, 600 University Avenue, Toronto, ON, M5G 1X5, Canada; 4 Sunnybrook Health Sciences Centre, 2075 Bayview Ave, Toronto, ON, M4N 3M5, Canada; 5 Centre for Addiction and Mental Health, 455 Spadina Avenue, Suite 302, Toronto, ON, M5S 2G8, Canada; 6 Department of Neonatology, Beth Israel Deaconess Medical Center, Rose 318, 330 Brookline Avenue, Boston, MA, 02115, USA

**Keywords:** Interpersonal psychotherapy, Postpartum depression, Randomized controlled trial

## Abstract

**Background:**

Substantial data indicate potential health consequences of untreated postpartum depression (PPD) on the mother, infant, and family. Studies have evaluated interpersonal psychotherapy (IPT) as treatment for PPD; however, the results are questionable due to methodological limitations. A comprehensive review of maternal treatment preferences suggests that mothers favor ‘talking therapy’ as a form of PPD treatment. Unfortunately, IPT is not widely available, especially in rural and remote areas. To improve access to care, telepsychiatry has been introduced, including the provision of therapy via the telephone.

**Methods/Design:**

The purpose of this randomized controlled trial is to evaluate the effect of telephone-based IPT on the treatment of PPD. Stratification is based on self-reported history of depression and province. The target sample is 240 women. Currently, women from across Canada between 2 and 24 weeks postpartum are able to either self-identify as depressed and refer themselves to the trial or they may be referred by a health professional based on a score >12 on the Edinburgh Postnatal Depression Scale (EPDS). Following contact by the trial coordinator, a detailed study explanation is provided. Women who fulfill the eligibility criteria (including a positive diagnostic assessment for major depression) and consent to participate are randomized to either the control group (standard postpartum care) or intervention group (standard postpartum care plus 12 telephone-based IPT sessions within 12 to 16 weeks, provided by trained nurses). Blinded research nurses telephone participants at 12, 24, and 36 weeks post-randomization to assess for PPD and other outcomes including depressive symptomatology, anxiety, couple adjustment, attachment, and health service utilization. Results from this ongoing trial will: (1) develop the body of knowledge concerning the effect of telephone-based IPT as a treatment option for PPD; (2) advance our understanding of training nurses to deliver IPT; (3) provide an economic evaluation of an IPT intervention; (4) investigate the utility of the EPDS in general clinical practice to identify depressed mothers; and (5) present valuable information regarding PPD, along with associated couple adjustment, co-morbid anxiety and self-reported attachment among a mixed rural and urban Canadian population.

**Trial registration:**

Current Controlled Trials Ltd. ISRCTN88987377.

## Background

Postpartum mood disorders are the most frequent form of maternal morbidity following childbirth [1]. These affective disorders range in severity from the early maternity blues to postpartum psychosis, a serious state affecting <1 % of mothers [2]. Within this group of disorders is postpartum depression (PPD), a condition often exhibiting the disabling symptoms of dysphoria, tearfulness, sleep difficulties, changes in appetite, guilt, anergia, problems with concentration, and suicidal ideation. Frequently exacerbating these indicators are low self-esteem, inability to cope, feelings of incompetence, and loneliness [3-6]. The onset rate of PPD is greatest in the first 12 weeks postpartum [7], with duration frequently dependent on severity [8] and time to onset of treatment [9]. While residual symptoms are common [10], 50 % of mothers will remain clinically depressed at 6 months postpartum [11,12], with an estimated 25 % of untreated PPD continuing past the first year [13].

PPD is a major health problem for many women from diverse cultures [14]. Longitudinal and epidemiological studies have yielded varying prevalence rates, ranging from 3 % to >25 % of women in the first year following delivery; these rates fluctuate due to sampling, timing of assessment, differing diagnostic criteria (major or minor depression), use of self-report measures, and whether the studies were retrospective (low rates) or prospective (6- to 10-fold higher). A meta-analysis of 59 studies reported an overall prevalence of major PPD to be 13 % [15]; it is noteworthy that the absolute difference in estimates between self-report assessments and diagnostic interviews was small, and rates did not differ between primiparous and multiparous mothers [15-17]. While the cause of PPD remains unclear [10], extensive research suggests a multifactorial etiology. Epidemiological studies and meta-analyses of predictive studies have consistently implied the importance of psychosocial variables [10,15,16], especially marital conflict [15,16,18-20] and a lack of social support [4,5,7,15,16,19,21].

PPD has well-documented health consequences for the mother, child, and family. While women who have suffered from PPD are twice as likely to experience future episodes of depression over a 5-year period [22], infants and children are particularly vulnerable. PPD can cause impaired maternal-infant interactions [23] and negative perceptions of infant behavior [24], which have been linked to childhood attachment insecurity [25,26], emotional developmental delay [25,27], and social/interaction difficulties [28,29]. Infants as young as 3 months of age are able to detect their mothers’ mood and to modify their own responses accordingly [30]. While cognitive skills [31], expressive language development [32], and attention [33] have been negatively influenced by PPD, children of depressed mothers are 2 to 5 times more likely to develop long-term behavioral problems [34,35]. Child neglect/abuse [36] and marital stress resulting in separation or divorce [37,38] are other reported outcomes. Maternal and infant mortality are rare but real consequences of PPD. Effective treatment of PPD is needed to not only help the mother but also to prevent these aforementioned consequences.

Antidepressant medication, cognitive behavioral therapy, and interpersonal psychotherapy (IPT) have been validated as effective treatments for general depression [39]. However, mothers are often reluctant to take antidepressants due to concerns about breast milk transmission or potential side effects [4]. Although there is evidence that antidepressants are relatively safe for breastfed infants, the American Academy of Pediatrics [40] classifies most antidepressants as ‘drugs whose effect on nursing infants is unknown but may be of concern’. Furthermore, recent studies have demonstrated several potential sequelae including cardiovascular malformations which have fueled the debate about the safety of antidepressants in the perinatal period [41,42]. Given these considerations, it is important that non-pharmacologic interventions be evaluated for use with postpartum women.

Dennis’ review of non-pharmacologic treatments [43] suggests that while psychosocial and psychological interventions may be beneficial, many studies have serious methodological limitations such as small sample sizes, non-random group allocation, or a lack of a control group. Thus, there remains a need for rigorous research to evaluate well-defined interventions for the treatment of PPD. To understand maternal treatment preferences for PPD, Dennis completed a qualitative systematic review [44]. For many mothers, the most desired treatment option was the opportunity to talk about their feelings [45-50]. In particular, the provision of ‘talking therapies’ with a health professional was universally expressed as the main treatment preference in a cross-cultural study incorporating 11 countries [51]. Maternal acceptance of treatment strategies is important due to its direct impact on intervention engagement and compliance [52,53]. Based on: (1) two meta-analyses suggesting that a lack of social support and marital conflict are consistent PPD risk factors [15,16], (2) maternal treatment preferences related to ‘talking therapies’ [51], (3) demonstrated effectiveness among depressed postpartum and non-postpartum samples [54-57], the evidence points to interpersonal psychotherapy (IPT) as a potentially effective treatment for mothers experiencing PPD.

IPT is a manual-based, time-limited psychotherapeutic approach with a basic premise that depression, regardless of etiology, is initiated and maintained within an interpersonal context. The goal of IPT is to achieve symptomatic relief for depression by addressing current interpersonal issues related to universal experiences of losses, changes, or conflicts associated with its onset or perpetuation; IPT does not seek to attribute interpersonal problems to underlying personality characteristics or unconscious motivations. IPT is primarily concerned with interpersonal functioning and symptoms, presumed to have biological, psychological, and social precipitants. There is a specific focus on social interactions, communication, social supports, and interpersonal functioning [58].

IPT is supported by principles of classic and contemporary interpersonal theories, including the works of Meyer, Sullivan, and Bowlby. Meyer conceptualized the bio-psycho-social formulation of illness [56]. Sullivan was among the first psychoanalysts to focus on interpersonal, rather than intrapsychic aspects of experience [59], and Bowlby was the father of attachment theory [60]. Attachment theorists view relationships as critical for survival and optimal functioning. When attachment relationships are ruptured, lost, or disordered, psychopathology can emerge, often in the form of depressive symptomatology and maladaptive interpersonal function. IPT can be seen as indirectly addressing these issues within the therapeutic frame [61]. IPT is also founded on empirical data based on the work by Brown and Harris on intimacy and social support [62,63], Pearlin and Lieberman on stressors including depression [64], and Weissman on the role of marital disputes in depression [65-67]. IPT sessions focus on examining the person’s interpersonal relationships, linking them to changes in mood, identifying a major problem area (that is, grief, interpersonal role disputes, role transitions or interpersonal deficits), relating symptoms to the problem area, and exploring alternative ways of being in relationships with others. Psychotherapeutic strategies used by the therapist include: seeking information, exploring parallels in other relationships, reviewing relationship and communication patterns, signaling what is significant, providing support, exploring affect, reviewing options, problem-solving, challenging maladaptive behaviors, and encouraging adaptive changes [68]. Generally, IPT consists of 12 to 20 weekly sessions lasting 45 to 60 min that may be provided by psychiatrists and a range of non-medical health professionals, including nurses [68-70].

Since its inception, IPT has been modified for different age groups (that is, adolescents to elderly) [71,72], types of mood disorders (that is, major depression, dysthymia, bipolar disorder) [69], and non-mood disorders (that is, bulimia, drug abuse, borderline personality disorder, social phobia, somatization, medically ill patients) [58]. It has also been used in a group format. Evidence from randomized controlled trials suggests that IPT is a reasonable alternative or adjunct to antidepressant medication as an acute, continuation, and/or maintenance treatment for patients with major depression [39]. In relation to the perinatal period, eight studies have evaluated the effectiveness of IPT for the treatment of antenatal depression and PPD.

In a US pilot study, 12 depressed pregnant women were offered eight weekly sessions of brief IPT followed by monthly maintenance sessions up to 24 weeks postpartum [73]. Nine participants completed the study with post-treatment data showing significant improvements in maternal mood, anxiety, and social functions. In another US pilot study, 13 depressed pregnant women were offered 16 weekly sessions of IPT [74]. Depression ratings decreased significantly throughout the treatment program and of the 10 women available for the 12-week post-treatment assessment, none reported depressive symptomatology using an Edinburgh Postnatal Depression Scale (EPDS) score >12. Building upon this pilot work, a 16-week randomized controlled trial was conducted with 50 low-income, Hispanic pregnant women who met DSM-IV criteria for depression; the mothers were randomly assigned to receive either IPT (*n* = 25) or a didactic parenting education program (*n* = 25) [75]. Thirty-eight (76 %) women were included in the data analysis. Depressed mood was measured with several instruments including the EPDS. The IPT group showed significant improvement compared to the parenting education control program on all measures of mood at post-treatment. Recovery criteria were met in 60 % of the women treated with IPT. These studies suggest IPT may be a promising intervention for antenatal depression.

IPT has also been evaluated for depression postnatally. In a single-group US study, six mothers with PPD were provided with 12 weekly sessions of IPT; significant improvements in mood were found post-treatment [76]. Advancing this preliminary work in a well-designed randomized controlled trial, 120 postpartum women meeting DSM-IV criteria for depression were recruited from the community and randomly assigned to either 12 weeks of IPT (*n* = 60) or a control group (*n* = 60) [77]. Follow-up data were collected via interview and self-report assessments of depressive symptomatology every 4 weeks; 99 (83 %) of the 120 women completed the protocol. Mean Hamilton Rating Scale for Depression (HRSD) scores of the women receiving IPT declined from 19.4 to 8.3, compared to just a 3-point decrease from 19.8 to 16.8 in the control group. Similarly, mean Beck Depression Inventory (BDI) scores of the women who received IPT declined from 23.6 to 10.6 over 12 weeks, compared to the control group decrease from 23.0 to 19.2. More women who received IPT recovered from their depressive episode based on HRSD scores ≤ 6 (37.5 %) and BDI scores ≤ 9 (43.8 %) compared with women in the control group (13.7 % and 13.7 %, respectively). However the outcomes assessors were not blinded to group allocation and the sample was very homogeneous (for example, primarily Caucasian, well-educated, and married).

IPT has also been evaluated in a group modality. In a single-group study, 17 Austrian mothers diagnosed with depression participated in 12 weekly sessions of group-based IPT [78]. Women also were provided with the telephone numbers of other group members to obtain additional support if needed. Mean score comparisons revealed significant changes from baseline to post-treatment for both the EPDS and HRSD. At post-treatment, 10 (59 %) mothers demonstrated full remission (HRSD < 9), 5 (29 %) established partial remission (score decrease >50 %), and 2 (12 %) showed no improvement. Follow-up assessments at 24 weeks post-treatment revealed a continued effect. However study limitations include the small sample size, absence of a control group, possible outcome assessment bias, and unknown level of intervention adherence. Furthermore, the possible effect of the co-intervention, telephone-based peer support, cannot be dismissed. Similar results were found in a small study of 18 depressed Australian mothers who were offered eight sessions of group-based IPT [79]. EPDS scores decreased pre- to post-treatment and the effect was maintained at the 12-week post-treatment follow-up. Limitations included the use of antidepressant therapy by 67 % of mothers and the lack of a control group. In a quasi-experimental study that included 36 US mothers diagnosed with depression, both mother-infant therapy and IPT were found to be superior to a waiting-list comparison group in reducing maternal depressive symptoms [80].

The connection between interpersonal relationships and psychological wellbeing is well established [81]; smaller social networks, fewer close relationships, and lower perceived adequacy of social support have all been linked to depressive symptomatology [81,82]. Joiner [83], in discussing the etiology of depression, noted that regardless of what other factors may be involved, the interpersonal context greatly affects: (1) whether a person becomes depressed, (2) the person’s subjective experience while depressed, and (3) the behavioral manifestations and resolution of the disorder. Based on a leading conceptual framework advanced by Cohen and Wills [84], there are several pathways through which interpersonal relationships can affect psychological wellbeing. Members of a social network may exert a salutary influence on mental health by providing normative guidance about health-relevant behaviors [85]. Integration in a social network may also directly produce positive psychological states, including a sense of purpose, belonging, and recognition of self-worth [86]. These positive states, in turn, may benefit mental health due to an increased motivation for self-care, as well as the modulation of the neuroendocrine response to stress [86]. Being part of a broader social structure (for example, involvement in social networks and immersion in close relationships) enhances the likelihood of accessing various forms of support, which in turn protects against distress [87]. Examples of such resources include access to health-relevant information or receipt of informal healthcare that could prevent a minor ailment from progressing into a more serious psychiatric disorder [86].

Interpersonal relationships may also act on several different points in the pathway between stressful events and eventual mental illness. First, the perceived availability of social support in the face of a stressful event may lead to a more benign appraisal of the situation, thereby preventing a cascade of ensuing negative emotional and behavioral responses [88]. Second, perceived or received support may either reduce the negative emotional reaction to a stressful event or dampen the physiologic/behavioral response to stress. Importantly, social support strategies to decrease depressive symptomatology have been described in the intervention literature [89,90] and through other descriptive studies [86,91].

Traditionally, IPT has been provided individually in a clinic setting or in a group modality. However, groups are poorly attended by new mothers [44], especially those who are feeling depressed; additional barriers to PPD treatment include stigma, lack of accessible treatment, time constraints, geographic barriers, and demands of child care [44]. Attrition rates in PPD clinic or group-based IPT trials are high, ranging from 20 % to 35 % [77,78]. An alternative to face-to-face healthcare contact is through telemedicine which has many advantages for diverse health problems [92-99] and has led some researchers to suggest that the telephone is perhaps one of the most under-utilized resources in healthcare [99]. Telephone-based interventions are not only flexible, private, and non-stigmatizing, but they also reduce differences related to socio-economic status and traditional healthcare barriers such as accessibility due to transportation or geography [98,100]. While in the last decade advances in technology, such as the utilization of email and the internet, have enhanced the range of options available for ‘home’ support, the telephone remains the most accessible to the majority of individuals [92].

Importantly, preliminary results suggest that IPT may be effectively provided via telephone. In a 12-week pilot randomized controlled trial to test the feasibility and efficacy of IPT delivered over the telephone, 30 depressed US women were randomized to receive either telephone-based IPT or no treatment [101]. Patients were interviewed at baseline and immediately post-treatment by a blind, clinical evaluator. Women in the intervention group, as compared to those in the control group, had significantly lower levels of depressive symptomatology as measured by the HRSD and improved social functioning as measured using the Social Adjustment Scale. These findings support the feasibility of providing IPT via telephone and that it may be an effective treatment for depression. Furthermore, 83 % of women were favorable toward the use of the telephone, 83 % saw the telephone as facilitating treatment, and 75 % wanted to continue their treatment via telephone. In relation to the treatment of PPD via telephone, only one small study has been published. In a US pilot test, 20 depressed mothers were provided with 10 weeks of ‘telecare’ administered by a nurse and consisting of modified cognitive behavioral therapy, relaxation techniques, and problem-solving strategies [102]. The BDI was administered pre- and post-treatment with scores significantly lower following treatment. Several other telephone-based interventions have been successfully used with new mothers [99], including Dennis’ Breastfeeding Peer Support Trial [103]. These studies are examples that suggest telephone-based interventions can positively influence maternal health outcomes including mood.

### Relevant systematic reviews

Five Cochrane systematic reviews have been completed related to PPD. Dennis evaluated the effect of psychosocial and psychological interventions for the prevention of PPD and found interventions may be more effective if they were individually based, initiated postnatally, and targeted mothers identified as ‘high-risk’ [104,105]. Dennis and Ross [106] evaluated the role of estrogen and progestogen in the prevention and treatment of PPD. Of the two included trials, progestogen was associated with an increased risk of PPD, while estrogen therapy had a modest value in the treatment of severe PPD. Hoffbrand [107] evaluated the effectiveness of different antidepressant medications, including comparisons to other forms of PPD treatment. The only included trial suggested that fluoxetine was just as effective as a course of cognitive-behavioral counseling in the short-term. However, > 50 % of eligible mothers refused participation in the trial, primarily due to their reluctance to take antidepressant medication. Barlow [108] evaluated the effect of group-based parenting programs on improving maternal psychosocial health including depression; the findings from the 23 trials suggested diverse parenting programs are effective in the short term. While the mean group drop-out rate was 28 %, several trials reported rates > 40 %; detailed analyses suggest mothers who were less educated, lower income, or an ethnic minority were more likely to withdraw. Dennis and Hodnett [109] conducted a systematic review of the effect of psychosocial and psychological interventions for the treatment of PPD. No studies have been found evaluating the effect of telephone-based IPT for PPD by any health professional.

### Research questions

#### Primary research question

What is the effect of telephone-based IPT by trained nurses on PPD at 12 weeks post-randomization (that is immediately post-treatment for mothers in the intervention group)?

#### Secondary research questions

What is the effect of telephone-based IPT by trained nurses on: (1) PPD at 24 and 36 weeks post-randomization (that is 12 and 24 weeks post-treatment for mothers in the intervention group; (2) depressive symptomatology at 12, 24, and 36 weeks post-randomization; (3) anxiety at 12, 24 and 36 weeks post-randomization; (4) couple relationship quality and adjustment at 12, 24, and 36 weeks post-randomization; (5) couple attachment at 12, 24, and 36 weeks post-randomization; and (6) health service utilization from randomization to 36 weeks post-randomization?

#### Other research questions

Other research questions are: (1) what are the cost implications of IPT versus usual care from a societal perspective; (2) what are mothers’ evaluations of their IPT experience; (3) what are nurses’ evaluations of their experience in learning and providing IPT; and (4) what are nurses’ reports of the type and intensity of their IPT activities?

## Methods/Design

### Main outcome measure

The primary outcome for this trial is PPD, as diagnosed by the Structured Clinical Interview for DSM-IV (SCID-I), administered at 12 weeks post-randomisation (that is immediately post-treatment for mothers in the intervention group).

### Intervention groups

#### Control group

Standard postpartum care as provided to all mothers, including community PPD services.

#### Treatment group

Standard postpartum care as provided to all mothers in addition to 12 weekly sessions of telephone-based IPT.

### Randomization procedure

Randomization is centrally controlled using a web-based randomization service, with stratification based on self-reported history of depression and province. This randomization service was successfully used by Dennis in the PPD prevention trial [110]. Provincial stratification is planned, because postpartum care is based on practice standards set by a province’s Ministry of Health. As such, ‘standard postpartum care’ will be very different for each province.

### Outcome measures

#### Primary outcome

##### Postpartum depression

The primary outcome for this trial is PPD, as diagnosed by the Structured Clinical Interview for DSM-IV (SCID-I) [111] administered at 12 weeks post-randomization (Table 1). The SCID-I is a semi-structured interview that includes an introductory overview followed by nine modules, seven of which represent the major DSM diagnostic classes. Because of its modular construction, the SCID-I can be adapted for use in studies in which particular diagnoses (for example, depression only) are of interest. As such, for this trial, the Current Major Depressive Episode (MDE) section of Module A will be used to indicate the presence of a major depressive episode and the Melancholic Features, Atypical Features, and Current Manic Episode sections of Module A will be used to further evaluate the severity of a potential participant’s mood episode(s) and the impact of these subcategories on a participant’s response to the IPT intervention.

**Table 1:** Timing of measurements

**Time**	**Outcome measures**
Baseline	(1) SCID-I, (2) EPDS, (3) STAI-State Anxiety Inventory, (4) Dyadic Adjustment Scale, (5) Experiences in Close Relationships-Revised, and (6) demographic variables
12 weeks post-randomization	(1) SCID-I, (2) EPDS, (3) STAI-State Anxiety Inventory, (4) Dyadic Adjustment Scale, (5) Experiences in Close Relationships-Revised, (6) Maternal Health Service Utilization and Cost of Care Questionnaire, (7) Maternal Satisfaction with IPT Questionnaire (to be completed by mothers in the intervention group), and (8) IPT Activity Log (to be submitted to the Data Coordinating Center by the IPT nurse)
24 weeks post-randomization	(1) SCID-I, (2) EPDS, (3) STAI-State Anxiety Inventory, (4) Dyadic Adjustment Scale, (5) Experiences in Close Relationships-Revised, and (6) Maternal Health Service Utilization and Cost of Care Questionnaire
36 weeks post-randomization	(1) SCID-I, (2) EPDS, (3) STAI-State Anxiety Inventory, (4) Dyadic Adjustment Scale, (5) Experiences in Close Relationships-Revised, and (6) Maternal Health Service Utilization and Cost of Care Questionnaire
Continuous	Nurse IPT Experience Questionnaire (to be completed either at the end of the trial or when an IPT nurse discontinues trial participation)

There are more than 500 reports of published studies using the SCID-I as a diagnostic instrument and the reliability and validity has been extensively reported in diverse studies and samples [112]. In particular, research evaluating the SCID-I has found the measure to yield highly reliable diagnoses for most disorders [112,113], with inter-rater reliability often exceeding 81 % for diagnoses such as major depression [114]. The SCID-I has also been effectively used with postpartum women to assess depression [115-120]. The SCID training materials, for both clinicians and research personnel, include a user’s guide and eight videotapes. At the UCLA Research Center for Major Mental Illnesses, a training and quality assurance program was used to evaluate SCID inter-rater reliability and diagnostic accuracy. Both clinically experienced interviewers and recently trained neophyte interviewers were able to achieve and maintain high levels of inter-rater reliability, diagnostic accuracy, and interviewer skill; at the first quality assurance check after training, there were no significant differences between experienced and neophyte interviewers in inter-rater reliability or diagnostic accuracy [121].

The SCID-I is also administered at 24 and 36 weeks post-randomization, as a secondary outcome. While some attrition of the sample is expected by this time, the additional information will provide important evidence of longer-term effects of the IPT intervention.

#### Secondary outcomes

##### Depressive symptomatology

This outcome is measured using the Edinburgh Postnatal Depression Scale (EPDS) [122], a 10-item self-report instrument. Items are rated on a 4-point scale to produce a summative score ranging from 0 to 30, with higher scores indicating lower maternal mood. This instrument does not diagnosis PPD but rather it is the most frequently used instrument to assess for postpartum depressive symptomatology [16]. Of the 30 PPD treatment studies found in the extant literature [43,123], 83 % (*n* = 25) used a self-report measure to evaluate whether the intervention was effective. Of these 25 studies, 18 (72 %) incorporated the EPDS. Created to counter the limitations of other well-established depression scales, it has been validated by standardized psychiatric interviews with large samples and has well-documented reliability and validity in over 11 languages [14]. The published recommended EPDS cutoff score of > 12 [122] is used to examine depressive symptomatology at 12, 24, and 36 weeks post-randomization.

##### Anxiety

This outcome is measured using the Spielberger State-Anxiety Inventory (STAI-State) [124], a 20-item self-report instrument developed to assess levels of relatively transient situation-related (state) anxiety. Items are rated on a 4-point Likert-type scale to produce a summative score ranging from 20 to 80 with higher scores indicating higher levels of anxiety. The STAI-State has been used widely in pregnancy and postpartum studies and anxiety has been consistently related to depressive symptomatology in new mothers [16].

##### Attachment

This outcome is measured using a revised version of Brennan, Clark, and Shaver’s Experiences in Close Relationships (ECR) questionnaire, the ECR-Revised (ECR-R) [125]. The ECR-R yields scores on two-dimensional subscales: attachment-related anxiety (that is fear of rejection and abandonment; items 1 to 18) and attachment-related avoidance (that is discomfort with closeness and discomfort with depending on others; items 19 to 36). Each item is rated on a 7-point scale that ranges from 1 (strongly disagree) to 7 (strongly agree). For the present trial, only those who report living with a spouse or partner in an intimate relationship complete this questionnaire.

##### Relationship quality and adjustment

This outcome is measured using the Dyadic Adjustment Scale (DAS) [126]. The DAS is a comprehensive, 32-item scale frequently used to measure adjustment in couple relationships, an aspect of relationship quality. Items are rated on Likert-type scales, with total DAS scores ranging from 0 to 150. A total DAS score may be derived by summing across the four subscales, each of which vary in length: dyadic consensus (items 1–3, 5, 7–15; score range 0 to 50), dyadic satisfaction (items 16–23, 31, 32; score range 0 to 24), dyadic cohesion (items 24–28; score range 0 to 65), and dyadic affectional expression (items 4, 6, 29, 30; score range 0 to 12) [126]. The DAS has demonstrated good psychometric properties with diverse populations and is commonly used in PPD studies [126,127]. Increased relationship quality and adjustment has been associated with decreased depressive symptomatology in postpartum women [127]. For this trial, only those who report living with a spouse or partner in an intimate relationship complete the DAS.

##### Health service utilization

This outcome is measured using a slightly modified version of the Health Service Utilization and Cost of Care Questionnaire [128], a self-report instrument developed and used by researchers at McMaster University’s Health and Social Service Utilization Research Unit. Previous research by Dennis [129] suggests that mothers with depressive symptomatology in the early postpartum period used significantly more health services than mothers with no depressive symptomatology.

#### Other outcomes

##### Maternal evaluation of IPT

This outcome is measured using the Maternal Satisfaction with IPT Questionnaire. Based on (1) Dennis’ previous maternal satisfaction questionnaires for the Breastfeeding [103] and PPD prevention trials [110,130], (2) the Client Satisfaction Questionnaire [131], and (3) the Patient Satisfaction Questionnaire [132], this outcome is assessed at 12 weeks post-randomization by the trial coordinator (who is not collecting any primary or secondary outcome data).

##### Nurse evaluation of IPT training and intervention

This outcome, assessed via telephone at the end of the trial, is measured using the Nurse IPT Experience Questionnaire. Based on similar content areas used in Dennis’ previous trials [103], questions are related to (1) IPT training, (2) supervision, (3) provision of IPT, and (4) personal effects.

##### Nurse IPT activities

All intervention activities (for example, telephone discussions, left messages, missed sessions) are documented by the IPT nurses using the IPT Activity Log. IPT nurses are requested to return the activity log for a mother immediately post-treatment. This activity log is similar to ones used in Dennis’ previous telephone-based trials [103,130], including the PPD prevention trial [110].

### Participants

#### Sample size

Two hundred and forty mothers >2 weeks and <24 weeks postpartum.

#### Justification for sample size

PPD psychosocial and psychological treatment trials have reported control group spontaneous recovery rates (EPDS < 13) ranging from 25 % to 40 % [130,133-136]. The difference in recovery rates between groups in these trials ranged from 27 % to 55 %, with 66 % to 85 % of mothers in the intervention group classified as non-depressed at the primary outcome time period. Among perinatal IPT trials (including both antenatal depression and PPD) where participant inclusion criteria included a clinical diagnosis of depression, reported control group spontaneous recovery rates (for example, HRDS < 7, BDI < 10) ranged from 15 % to 18.3 % [75,77]. The difference in recovery rates between groups in these trials ranged from 16.7 % to 44.6 %, with 32 % to 60 % of mothers in the intervention group classified as non-depressed at the primary outcome time period. These results should be interpreted with caution, due to the different methods of measuring depressive symptomatology and small sample sizes. In the most rigorous IPT trial for PPD (*n* = 120), 38.3 % of mothers in the IPT group and 18.3 % of mothers in the control group were classified as non-depressed immediately post-treatment (that is 12 weeks post-randomization), a 20 % difference [77].

Given that (1) all the preceding studies used self-report measure to assess recovery (for example, no depressive symptomatology using a specific cutoff score) instead of a diagnostic interview such as the SCID, and (2) the proposed trial’s sample will be heterogeneous and include rural and urban mothers from diverse provinces, for the purpose of calculating the sample size, we have made the conservative assumption that 30 % of mothers in the control group will spontaneously recover and will not have major depression at the 12 week post-randomization assessment (70 % will remain clinically depressed) (Table 2). The sample size is based on the ability to detect a moderate decrease of 20 percentage points to a rate of 50 % among mothers in the intervention group. Our partners in the participating public health departments agree that a 20 % absolute decrease in the number of mothers with depression post-treatment is the minimum effect that would be considered clinically meaningful, warranting the time and effort to develop and implement an IPT program. A two-tailed α error of 0.05 was chosen, as there is insufficient empirical evidence of either risk or benefit associated with the proposed telephone-based IPT intervention. Thus, with 80 % power, a two-tailed α error of 0.05, and using a test of two independent proportions to find a 20 % reduction in the number of mothers with depression at 12 weeks post-randomization (immediately post-treatment for mothers in the intervention group), a sample size of 188 (94 per group) is required; we plan to enroll 240 to allow for up to 20 % loss to follow-up. This sample size also provides power to detect a 20 % absolute reduction in other secondary outcomes, such as anxiety.

**Table 2:** Sample size differences between groups

**Control group depression rate**	**IPT group depression rate**	**Size per group**
75	55	88
70	50	94
5	45	96

#### Inclusion criteria

Inclusion criteria are: (1) live birth; (2) mother 2 to 24 weeks postpartum; (3) infant discharged from hospital and at home with mother; (4) clinical diagnosis of major depression using the SCID-I; and (5) understands spoken English. Due to the nature of the intervention, it is not feasible to recruit mothers unable to speak English.

#### Exclusion criteria

Exclusion criteria are: (1) current use of antidepressant or antipsychotic medication; (2) currently receiving any form of individual psychotherapy administered by a trained professional; (3) active suicidal or self-harm thoughts; (4) current or past manic depression or bipolar diagnosis; and (5) chronic depression (episode length > 2.0 years). These exclusion criteria are consistent with other IPT trials [71,72,77].

### Trial process

#### Referrals

Referrals for the trial come from a variety of sources including public health departments, mental health units, and private practice clinics.

#### Public health

Currently, 24 public health departments across Canada (Ontario, Manitoba, Saskatchewan, and British Columbia) are assisting with recruitment for this trial. In Canada, public health departments receive notification of women who have given birth in their health region. As part of standard practice, each mother receives a telephone call or home visit from a public health nurse within the first few weeks postpartum following hospital discharge. During these interactions, public health nurses often administer the EPDS to assess maternal mood. A brief description is provided to mothers who are identified as experiencing depressive symptomatology (EPDS > 12) after the first 2 weeks postpartum (in order to avoid false-positives that are ‘maternity blues’). If verbal consent is obtained, the potentially eligible mother’s contact information is sent to the trial coordinator.

#### Other health professionals

In addition to referrals from public health departments, health professionals in public or private practice across Canada may also refer potentially eligible mothers.

#### Self-referrals

Women who are between 2 and 24 weeks postpartum and self-identify as feeling down or depressed may also self-refer to the trial.

All referred mothers are contacted by the trial coordinator and provided a detailed explanation of the study. If the mother expresses interest in participating in the trial, she is further assessed for eligibility. Eligible women are asked to provide verbal consent to participate in the study; if she consents, baseline information is collected by the trial coordinator. Using a web-based, centrally controlled randomization service, the mother is randomized to either the control (standard postpartum care) or intervention group (standard postpartum care plus 12 telephone-IPT sessions), with stratification based on province and self-reported history of depression. Following randomization, participants are immediately informed of their study group assignment.

#### Consent

Two copies of the study explanation and participant consent form are mailed to each participant. A postage-paid, addressed envelope is included in the mailed package so that one copy of the signed consent form is returned to the trial coordinator and the other copy is retained by the participant for her own records. These informed consent procedures have been used effectively by Dennis in previous trials [110,130].

With detailed informed consent procedures, it is expected that mothers will accept their group allocation following randomization. While most health professionals caring for participants and research nurses will be blinded, mothers cannot be. However, a formal telephone-based IPT PPD program does not already exist in the participating health regions, thereby ensuring no mother in the control group will receive the intervention. There will be no interference with usual medical/nursing care, which for some mothers may include antidepressant medication and/or psychotherapy. Very few mothers will have available and receive psychotherapy. As such, the actual number of trial participants in the control group who will receive psychotherapy will be very small; data about the presence of psychotherapy external to the trial will be collected during the health service utilization assessment. Information about interventions to treat PPD will be obtained from the participants and considered in the data analyses.

#### Treatment

Mothers allocated to the treatment group are contacted by the IPT nurse within 72 h of trial randomization and then weekly thereafter for 12 sessions.

#### Post-randomization data

To assess compliance to IPT, all intervention activities (for example telephone discussions, left messages, missed sessions) are documented by the IPT nurses using the IPT Activity Log. IPT nurses are requested to return the activity log for a mother immediately post-treatment.

#### Follow-up

**Figure 1:**
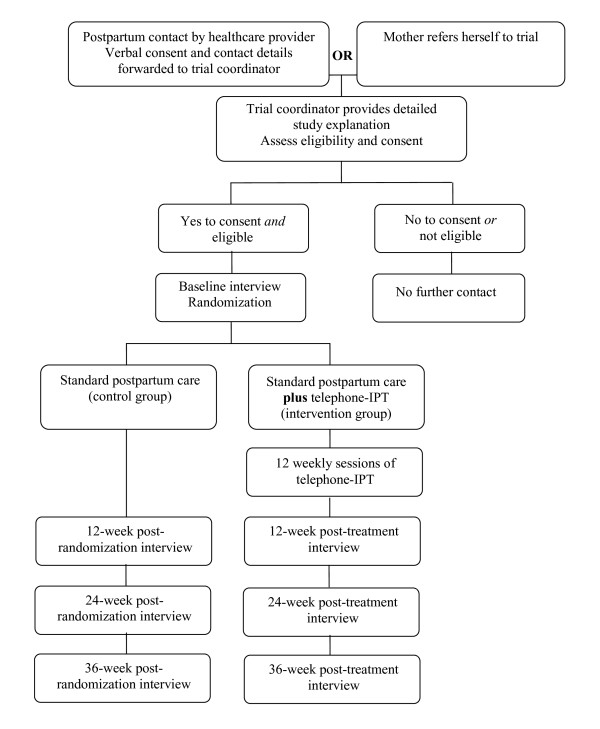
Trial Process.

Research nurses blinded to participant group allocation telephone study participants at 12, 24, and 36 weeks post-randomization/post-treatment to determine trial outcomes. PPD at 12 week post-randomization was chosen as the primary outcome because (1) we desire to provide comparable results to other perinatal IPT trials [73-80], and (2) the likelihood of losses to follow-up increase over time. So as to (1) address criticisms of previous trials related to insufficient follow-up to determine the longer-term effects of IPT and (2) present comparable results to other PPD treatment trials [137,138] including IPT trials [77,78], participants are contacted again at 24 and 36 weeks post-randomization and assessed for PPD and other outcomes (Figure 1).

All outcome data are administered over the telephone and entered into a Microsoft Access database with built-in logic and range checks to allow for immediate correction of errors and insertion of missing data.

#### Ethical approving body

This research protocol has been granted full ethical approval by the University of Toronto Health Sciences Research Ethics Board.

### Recruitment rate

The participant recruitment rate for this trial is founded on three factors: (1) the ability of public health nurses and other health professionals to identify and refer mothers with depressive symptomatology (EPDS > 12), (2) the number of referred mothers diagnosed with major depression (based on SCID interview), and (3) participant acceptance of the trial. Based on the health regions’ birth rates, the assumption that approximately 13 % of mothers will experience PPD [15], and that less than 50 % of mothers seek treatment for PPD [44], we conservatively estimate that 20 % of depressed mothers will be detected by public health nurses and other health professionals each year. In one IPT trial that initially identified mothers with a self-report measure (for example EPDS > 12) before applying a clinical diagnosis (for example SCID), only 65 % of women with depressive symptomatology were diagnosed with major depression [78]. In another IPT trial, 64 % of patients identified with depressive symptomatology (for example CES-D) were diagnosed with major depression [139]. Research has demonstrated high maternal acceptance to completing the EPDS [130] and both of Dennis’ breastfeeding [103] and PPD prevention trials [110,130] had good acceptance rates (> 66 %) to postpartum telephone-based interventions. Furthermore, in a review of maternal treatment preferences completed by Dennis, depressed mothers clearly indicated that they preferred ‘talking therapies’ [44]. For PPD IPT trials that were either clinic or group-based, acceptance rates of eligible mothers ranged from 51 % [77] to 68 % [79]. As such, we expect to recruit our sample size of 240 mothers with major depression in 2.5 years based on: (1) a public health department 20 % identification rate of mothers with EPDS scores > 12, (2) a conservative 50 % clinical diagnosis of major depression rate, (3) a 50 % maternal acceptance rate, and (4) a 15 % exclusion rate due to maternal use of antidepressants.

### Operational details

#### Telephone-IPT intervention

Mothers allocated to the treatment group receive 12 weekly IPT sessions, each session 50 to 60 min in duration and delivered over the telephone by a trained IPT nurse who receives weekly supervision by an IPT trainer (PR and SG). Within 72 h, the IPT nurse will either administer session 1 (if possible) or at least make an initial call to the participant to schedule session 1 (to occur within the week following enrolment). The scheduling of IPT sessions are based on mutual availability between the mother and IPT nurse. IPT is administered according to the standardized manual by Stuart and Robertson [56,140], with some modifications to accommodate the postpartum context of depression. These modifications are based on previous IPT trials for PPD [141].

As conducted in standard IPT clinical practice, a minority of trial participants identified as either in acute crisis at the end of treatment, or who are progressing however remain significantly symptomatic, thus would benefit from extending the course, may be offered up to four additional IPT sessions (that is, conduct 12 or a maximum of 16 sessions per trial participant, as appropriate). A full course of therapy must be completed within 16 weeks to account for any additional sessions and/or occasional scheduling conflicts or difficulties.

The initial sessions (1 to 3) will be concerned with placing the depression in an interpersonal context, reviewing the mother’s current and past interpersonal relationships, and relating problematic aspects of their relationships to the mother’s current depression. The IPT nurse and mother will collaboratively identify the problem area(s) most related to the episode and set treatment goals. During the intermediate sessions (4 to 9), the nurse will focus on the interpersonal difficulties identified by the mother, and utilize the IPT therapeutic guidelines to work through these focal problems. Based on previous IPT trials [77,141], common postpartum IPT problem areas include conflict with partner or extended family (interpersonal disputes), loss of social/work relationships (role transition), and losses unassociated with the birth, such as the death of a significant other (grief). In the final sessions (10 to 12), the nurse will reinforce the mother’s progress and efforts, her sense of competence in overcoming depression, consolidate her gains, discuss feelings or worries about concluding therapy, and work with the mother to develop plans should the depression recur. All sessions are audio-taped for supervision and monitoring purposes.

#### IPT training

Recruited nurses undergo extensive IPT training as lead by highly skilled and experienced IPT supervisors, Ravitz and Grigoriadis (also psychiatrist co-investigators) comprised didactic learning and supervised clinical practice. Previous studies have demonstrated that nurses can be effectively trained to provide IPT [68-70]. Prior to trial initiation, IPT nurses read and become familiar with the Interpersonal Psychotherapy manual (by Stuart and Robertson) and the postpartum modifications based on previous PPD IPT trials [141]. Each IPT nurse attends a didactic workshop with videotape presentations (minimum 5 h duration), meeting the standard for training of IPT therapists.

In addition to attending a workshop and reading about IPT, study nurses must successfully complete two supervised IPT clinical cases of 12 sessions each with patient volunteers who have been diagnosed by referring psychiatrists with PPD. The first case is done in person, and the second case is conducted by telephone following one initial face-to-face meeting with the nurse. All sessions are audio-recorded and reviewed in full by the supervisors who provide weekly supervision and feedback on the clinically applied principles and therapeutic techniques of IPT. An IPT nurse is considered ‘fully trained’ and prepared to administer the intervention to enrolled trial participants when she demonstrates a high degree of therapeutic competence and adherence (as monitored by IPT supervisors) and successfully completes IPT administration to the two test cases.

#### IPT supervision

While some researchers suggest that IPT therapists who perform well on their first supervised case do not require further intensive supervision [142], to ensure ongoing treatment fidelity, all fully trained and trainee IPT nurses are required to participate in weekly group supervision meetings. IPT nurses are also able to contact intervention supervisors as necessary to address immediate concerns.

#### IPT rating

Audio-recorded IPT sessions are randomly reviewed by an independent and IPT-trained rater to document treatment fidelity using the IPT Adherence Checklist. Adherence raters are trained to achieve > 90 % agreement with one of the IPT expert trainers.

#### Compliance

It is expected that 80 % of the intervention group will receive IPT due to the specific inclusion/exclusion criteria, informed consent procedures, and intervention compliance procedures. Some of the participants will drop out because of their preference for in-person therapy, scheduling difficulties, unsuccessful contact post-randomization or due to crises that can in some cases require contact with child protection service agencies, public health and/or their family physicians. In this trial, treatment compliance means the completion of at least 10 IPT sessions. Monitoring compliance of the intervention are three-fold: (1) IPT nurses’ completion of IPT Activity Logs for each participant, (2) supervision of the IPT intervention as provided by Ravitz and Grigoriadis, and (3) mothers’ evaluations of their IPT experiences.

#### Loss to follow-up

Maternal questionnaires were designed to require low levels of literacy and relatively little time to complete; they have been used previously with postpartum mothers and examined for clarity, comprehension, and ease of use. In particular, all measures (except the DAS and ECR-R) were used as outcome measures in Dennis’ PPD prevention trial with no difficulty. Attrition is a significant threat to both internal and external validity in intervention and longitudinal studies, as the average retention rate of 6-month to 2-year follow-up studies has been estimated at 62 % [143]. The following proactive telephone strategies [144] are employed to encourage high retention rates. Each questionnaire contains a contact form for the IPT nurse to record the date, time of call, status of call (for example busy, call back, no answer, and so on), and comments. Participants are telephoned during their stated’best time to contact’ period, surveys are cycled up to three times per day, and if possible, messages are left. Directory assistance is telephoned for all disconnected and wrong numbers. The desired goal is a minimum response rate of 90 %. Additional strategies to ensure a low attrition rate, based on a Cochrane review [145] and other research [146], are as follows: (1) obtaining a secondary contact number (such as the participant’s mother) from each participant at recruitment, (2) conducting telephone reminder calls of impending interviews, and (3) providing a small token of appreciation provided to all mothers who complete the 36-week post-randomization interview. Similar strategies were used in Dennis’ PPD prevention trial [110] with good results (retention rate = 87 % at 12 week assessment and 85 % at 24 week assessment).

### Statistical analysis plan

#### Primary analysis

Data will be analyzed using SAS software and an intent-to-treat approach will be used. Every effort will be made to collect follow-up data from all participants. We will not impute a score for missing primary outcome data. A two-sided significance level of 0.05 will be used for the primary outcome; a significance level of 0.01 (two-sided) will be used for secondary and other outcomes to account for multiple comparisons. The demographic and other baseline variables will be compared between study groups using descriptive statistics (means, standard deviations, proportions). For the primary analysis, a two sample two-sided test of proportions will be used to compare the difference in the incidence of PPD (major depression using the SCID) at 12 weeks post-randomization between the intervention and control groups. For secondary analyses, the statistical method to compare groups will depend on the distribution of the outcome variable in question. For binary variables (for example EPDS score > 12), a contingency table chi-square for a 2 × 2 table will be calculated and presented together with estimates for the relative risk, relative risk reduction, absolute risk reduction, and the number needed to treat. The corresponding 95 % confidence intervals for each estimated parameter will also be provided. Covariate variable adjustment will be achieved using logistic regression. For ordered categorical variables, a chi-square for trend will be calculated. Relative risks for each category versus a base category will be presented. Continuous variables with repeated measurement (such as anxiety) will be first analyzed using a single, repeated measures MANOVA consisting of group and time factors to test for the study’s overall intervention, time, or interaction effect. Following the detection of a significant effect, separate repeated measures 2-factor analyses of variance (RANOVA) with appropriate contrasts will show the nature of the effect within each outcome variable. Covariate variable adjustment will be achieved using analysis of covariance and linear regression. Finally, the relationship between the number of IPT sessions on PPD, and other study outcomes will be assessed to determine an effective dose response.

#### Economic evaluation

The proposed intervention may have economic implications through two mechanisms. First, there may be differential personnel and administrative costs related to the IPT program itself. Second, pilot data suggest that the intervention may lead to decreased use of healthcare resources [130]. The objective of the economic analysis will be to estimate the cost-effectiveness of the addition of telephone-based IPT compared to standard postpartum care of mothers with PPD. In order to facilitate comparison with other healthcare programs, the analysis will take a societal perspective, in which consideration will be given to all costs regardless of the parties to whom they accrue. Thus, direct medical costs will be supplemented by data on volunteer labor costs, medical and non-medical costs borne by the mother herself or her family, and mother or family time absent from work. Cost data will also be presented in a disaggregated format to improve the generalizability of the results to parties having other perspectives, such as the provincial ministry of health or community organizations.

#### Comparators and time horizon

The alternatives in the trial are telephone IPT vs. standard postpartum care of mothers with PPD. Given that IPT in this context is adjunctive, the economic analysis will retain a high degree of external validity. The economic component of the study will measure costs from randomization to 36 weeks post-randomization. We recognize that the resource implications of the trial intervention will probably extend beyond that point. As the incidence of affective symptoms gradually decreases, however, the longer-term cost differences between programs are also likely to decrease. Moreover, the costs of implementing the intervention itself will be fully captured. The measured cost-effectiveness will therefore be a conservative estimate. Decision analytic modeling of longer-term outcomes beyond 36 weeks will be undertaken in secondary analyses if there are still differences between groups in resource utilization and costs at the end of trial data collection.

#### Analytic technique

Results will be approached using incremental cost-effectiveness analysis, in which the additional costs and effects for the IPT will be compared to the costs and effects of standard community postpartum care for mothers with PPD. All analyses will be performed on an intention-to-treat basis.

#### Measurement of resource utilization

We anticipate that resource use will consist of the following factors: (1) direct medical costs, including (a) fees for healthcare professionals (b) hospital care, (c) drug prescriptions (anti-depressant medication); (2) direct non-medical costs, including (a) travel costs incurred by patients to obtain care, (b) child care expenses; (3) productivity losses due to patient and family work absence; and (4) cost to administer the IPT intervention including (a) recruiting and training nurses, (b) nurses’ time for providing IPT, and (c) IPT supervision.

Resource use will be ascertained through activity logs completed by the IPT nurses. For the use of professional healthcare services, participants will complete a slightly modified version of the self-report Health Service Utilization and Costs of Care Questionnaire [128]. The latter questionnaire also includes items for direct non-medical costs and work absence.

#### Valuation of unit costs

Physician fees will be derived from local provincial fee schedules [147] and average hourly wages for registered nurses will be applied. Price weights for hospitalization episodes will be obtained using aggregate data from standardized hospital cost accounting systems in British Columbia and Alberta and from the Ontario Case Cost Project of the Ministry of Health Joint Policy and Planning Committee [148]. Total costs per patient will be the summed products of quantities of resources used multiplied by these unit prices. Given that the time horizon for the measured outcomes included in the current study protocol is short, discounting of costs and effects will not be undertaken for the primary analysis.

#### Determination of effects

Efficacy will be expressed as the recovery of PPD at 12 weeks post-randomization (immediately post-treatment for mothers in the intervention group), as dictated by the primary outcome of the trial. Secondary cost-effectiveness ratios consistent with the trial’s secondary endpoints will also be reported. Given the relatively standardized nature of the intervention and the acute setting, compliance is anticipated to be good. Furthermore, the study population is not very restrictive, with the only excluded patients being those already receiving pharmacological or psychotherapeutic intervention, those with a current or past bipolar diagnosis, and/or those with chronic depression or suicidal ideation, most of whom would not avail themselves of the intervention outside of the trial context. It is likely therefore that the economic evaluation will have very good external validity; no adjustments to approximate real-world effectiveness are planned.

#### Budgetary impact analysis

In addition to the preceding efficiency analysis, the budgetary impact at the participating health regions will be explored. In this analysis, the total personnel costs for each of the health units will be compared, assuming that health units either persist with standard postpartum care for mother with PPD or alternatively adopt the telephone-based IPT program. This analysis will assume that the care of new mothers with PPD had costs and outcomes of either the intervention or control group. The analysis will be repeated under several assumptions regarding the size of the program, the percentage of depressed mothers identified, and the percent of a nursing full-time equivalent required to provide and maintain the IPT program.

#### Uncertainty and statistical issues

Uncertainty will be assessed through sensitivity analysis for non-stochastic inputs such as choice of price weights. For stochastic variables including efficacy and costs, a non-parametric bootstrap method will be used to generate joint confidence intervals for the cost-effectiveness ratio. This technique involves resampling, with replacement, of the observed distribution of costs and effects, to establish an empirical distribution of cost-effectiveness ratios, from which confidence intervals can be constructed. Sample size will be determined by statistical consideration for the detection of efficacy in the clinical trial. In the absence of information regarding the dependence of costs and effects, it is problematic to determine power for comparisons of cost-effectiveness ratios a priori. The economic evaluation will therefore take an estimation, rather than hypothesis testing, approach to the construction of incremental cost-effectiveness ratios. Cost-effectiveness ratios and 95 % confidence intervals will be estimated using non-parametric bootstrapping [149]. The frequency with which these intervals overlap with cost-effectiveness thresholds of $0 to $300,000 per PPD recovery will be reported using cost-effectiveness acceptability curves.

#### Exploratory analyses

Exploratory analyses will be performed using data collected in the entire sample to explore relationships among the SCID and other study outcomes.

#### Predefined subgroups

None.

### Trial Steering Committee

Members of the Steering Committee include the PI (Dennis), co-investigators (Ravitz, Grigoriadis, Hodnett, Ross, Zupancic), trial coordinator (Jovellanos), and biostatistician (Kiss).

### Data monitoring and Ethics Committee

Judith Lumley and Donna Stewart.

### Trial status

Recruitment is ongoing with 212 participants enrolled as of March 9, 2012.

## Abbreviations

BDI Beck: Depression inventory; CES-D: Centre for epidemiologic studies depression scale; DAS: Dyadic adjustment scale; DSM-IV: Diagnostic and statistical manual of mental disorders; ECR: Experiences in close relationships; ECR-R: Experiences in close relationships - revised; EPDS: Edinburgh postnatal depression scale; HRSD: Hamilton rating scale for depression; IPT: Interpersonal psychotherapy; MDE: Major depressive episode; PPD: Postpartum depression; SCID-I: Structured clinical interview for dsm axis i and ii disorders; STAI-State: Spielberger state-anxiety inventory.

## Competing interests

The authors declare that they have no competing interests.

## Authors’ contributions

C-LD conceived of the study and developed the background, methods and design. She also wrote the grant to obtain funding. She drafted the manuscript, critically revised it for important intellectual content, and provided final approval of the submitted version. Currently, she provides overall trial management and staff supervision. PR and SG contributed to the development of the trial’s IPT intervention and helped to draft and critically revise the intellectual content of the manuscript. They also provided final approval of the submitted manuscript. Currently, they lead IPT supervision, monitoring, training, and adherence rating. MJ provides day-to-day, overall trial management, coordinating training and supervising staff, recruiting participants, and acquiring baseline data. She assisted in drafting and revising the manuscript and provided final approval of the submitted version. EH reviewed the trial methods for the grant proposal. She provided approval on the final version of the manuscript. LR contributed to study design, manuscript revisions, and provided approval of the final manuscript. JZ contributed to the economic evaluation plan and provided final manuscript approval. All authors read and approved the final manuscript.

## Authors’ information

C-LD (PhD) is an Associate Professor at the University of Toronto and Senior Scientist at the Women’s College Research Institute in Toronto, Canada. She holds a Canada Research Chair in Perinatal Community Health at the University of Toronto and the Shirley Brown Chair in Women’s Mental Health Research at Women’s College Research Institute. PR (MD) holds the Morgan Firestone Psychotherapy Chair and is Head of the Psychotherapy Program and an Associate Professor in Psychiatry for the University of Toronto. She is also Director of the Mount Sinai Hospital Psychotherapy Institute in Toronto, Canada. SG (MD, PhD) is an Associate Professor in Psychiatry at the University of Toronto and a Canadian Institutes of Health Research (CIHR) New Investigator. She is Fellowship Director in the Department of Psychiatry at the University Health Network and Academic Leader for the Reproductive Life Stages program at Women’s College Hospital. MJ (MSc) is Research Manager for the Mothering Transitions program at the University of Toronto. She coordinates all research-related activities and provides personnel supervision and coordination of staff training within the Mothering Transitions program. She is a member of the Society for Clinical Trials and Clinical Research Association of Canada. EH (RN, PhD) is a Professor at the Lawrence S. Bloomberg Faculty of Nursing, University of Toronto. LR (PhD) is an Associate Professor in Psychiatry at the University of Toronto and Senior Scientist at the Center for Addictions and Mental Health, Toronto, Ontario JZ (MD, PhD) is an Assistant Professor of Pediatrics at the Beth Israel Deaconess Medical Center, Boston, Massachusetts, USA.
